# Orthogonal shortwave infrared emission based on rare earth nanoparticles for interference-free logical codes and bio-imaging†
†Electronic supplementary information (ESI) available. See DOI: 10.1039/c8sc05044a


**DOI:** 10.1039/c8sc05044a

**Published:** 2019-01-24

**Authors:** By Liyi Ma, Xuejiao Zhai, Gaiping Du, Jing Zhou

**Affiliations:** a Department of Chemistry , Capital Normal University , Beijing 100048 , People's Republic of China; b Key Laboratory of Photochemical Conversion and Optoelectronic Materials , Technical Institute of Physics and Chemistry , Chinese Academy of Sciences , Beijing 100190 , People's Republic of China; c University of Chinese Academy of Sciences , Beijing 100049 , People's Republic of China . Email: jingzhou@cnu.edu.cn

## Abstract

Shortwave infrared (SWIR) photoluminescence has received intense interest in many fields in recent years thanks to the advantages of its wide wavelength range and high tissue imaging ability and it is invisible to the naked eye. However, achieving orthogonal SWIR emission still remains a challenge. In the present study, synthesized NaErF_4_@NaLuF_4_ (Er@Lu) and NaYF_4_:Nd@NaLuF_4_ (Y:Nd@Lu) nanoparticles emitted atom-like SWIR emission, and the separation distance between the SWIR emission was beyond 50 nm, which permitted orthogonal SWIR signal acquirement with optical filters. Furthermore, an invisible logical code was designed by manipulating the orthogonal SWIR emission of the lanthanide fluoride nanoparticles, and was further operated by basic logical operations and applied in information encryption and anti-counterfeit fields. In addition, the emission between these two hydrophilic nanoparticles could also be separated *in vivo* without signal interference and the orthogonal SWIR imaging mode was achieved, which was demonstrated in a bio-imaging experiment *in vivo*. This demonstration extended the orthogonal SWIR emission capacity by controlling the orthogonal emission, opening new opportunities in the fields of data security, disease diagnosis and non-interference label *in vivo*.

## Abstract 2

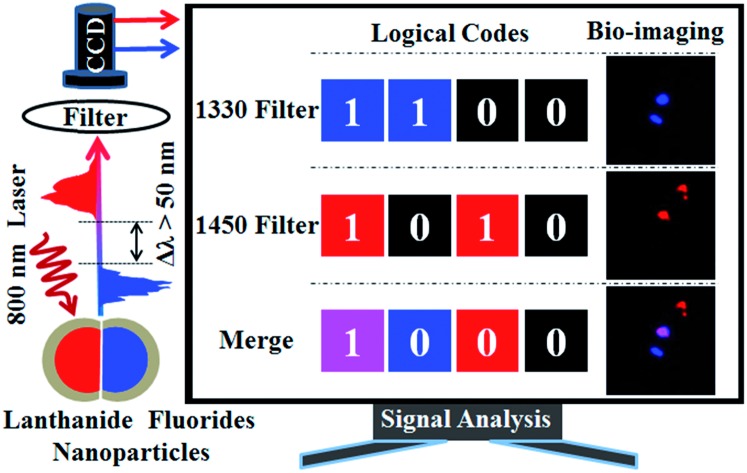
The NaErF_4_@NaLuF_4_ and NaYF_4_:Nd@NaLuF_4_ emitted orthogonal shortwave infrared (SWIR) lights, which were separated by optical filter, applied in invisible logical codes and interference-free bio-imaging.

## Introduction

Many photoluminescence materials have been investigated to precisely tune the wavelength,^1^ lifetime,^2^ and polarization of emitted light,^3^ and applied in many fields, including bio-imaging^1^,^4^ and anti-counterfeit.^5^ Tuning the lifetime and polarization of photoluminescence has some inherent problems, such as a shortage of material diversity, expensive equipment, a long period required to collect signals and signal overlap.^2^,^6^,^7^ However, tuning the emission wavelength is easily achieved not only on materials but also instruments.^1^,^8^ Orthogonal emission spectra systems are urgently required to simultaneously obtain accurate multipath signal identification in many applications, where signal interference between two or more photoluminescence materials can be directly ignored, due to completely separated emission spectra.^9^

Recently, shortwave infrared (SWIR) photoluminescence has attracted considerable attention in the assessment of new SWIR materials and their applications, due to its unique advantages, such as a wide wavelength range (1000–2300 nm), and it is invisible to the naked eye and has excellent tissue imaging ability.^10^–^16^ These studies primarily focus on discovering novel materials and “single-color” imaging *in vivo*, but, these are emerging urgent demands to achieve new applications and simultaneous multi-channel SWIR imaging for various concerned objects.^6^,^7^,^9^ It is reported that dyes,^17^–^19^ quantum dots (QDs),^9^,^20^ conjugated polymers,^21^ aggregation-induced-emission (AIE) materials^22^ and single-wall carbon nanotubes (SWCNTs)^23^ have the ability to tune emission wavelength by precisely adjusting the structure, size, or composition of the materials. However, the emission spectra generated by these SWIR probes could not be easily isolated, which would limit the diversity of labels in the SWIR region and their application in simultaneous multi-channel imaging in bio-imaging. On the other hand, lanthanide ions have a unique 4f electron configuration, which provides these ions with unique optical properties and atom-like emission.^24^,^25^ Tunable SWIR emission of lanthanide fluoride nanoparticles (Pr^3+^, Ho^3+^, Er^3+^, and Tm^3+^ doping) is achieved, but a 980 nm laser matches the absorbance peak of water, which can induce tissue hyperthermia and easily injure normal tissues, and is thus an obstacle in its application in practice.^24^ Dye-sensitized multi-shell lanthanide fluorides excited by an 800 nm laser (alternative excitation laser) can emit tunable SWIR light, but the dye has poor photo-stability and obvious visible color that limit long-term observation and the invisibility of lanthanide fluoride probes in some fields.^26^ Therefore, achieving photostable orthogonal SWIR emission excited by an 800 nm laser still remains a great challenge. It is known that lanthanide nanoparticles containing Nd^3+^ or Er^3+^ can emit SWIR signals, which can be excited by an 800 nm laser.^27^–^30^ Detailed analysis of these spectra showed that an essentially orthogonal SWIR emission between Nd^3+^ (1290–1384 nm) and Er^3+^ (1448–1656 nm) was found without signal interference. This orthogonal emission property would permit simultaneous multi-channel SWIR imaging with interference-free signals, which could be further applied in many fields.

In the present study, NaErF_4_@NaLuF_4_ (Er@Lu) and NaYF_4_:Nd@NaLuF_4_ (Y:Nd@Lu) were designed and synthesized. Doping Nd^3+^ or Er^3+^ ions emitted completely separated SWIR spectra, which were excited by an 800 nm continuous laser. Furthermore, orthogonal SWIR emission was achieved using optical filters without interference, and for the first time their applications were investigated in invisible logical codes for information encryption, anti-counterfeit and orthogonal SWIR imaging *in vivo*.

## Results and discussion

**Fig. 1:**
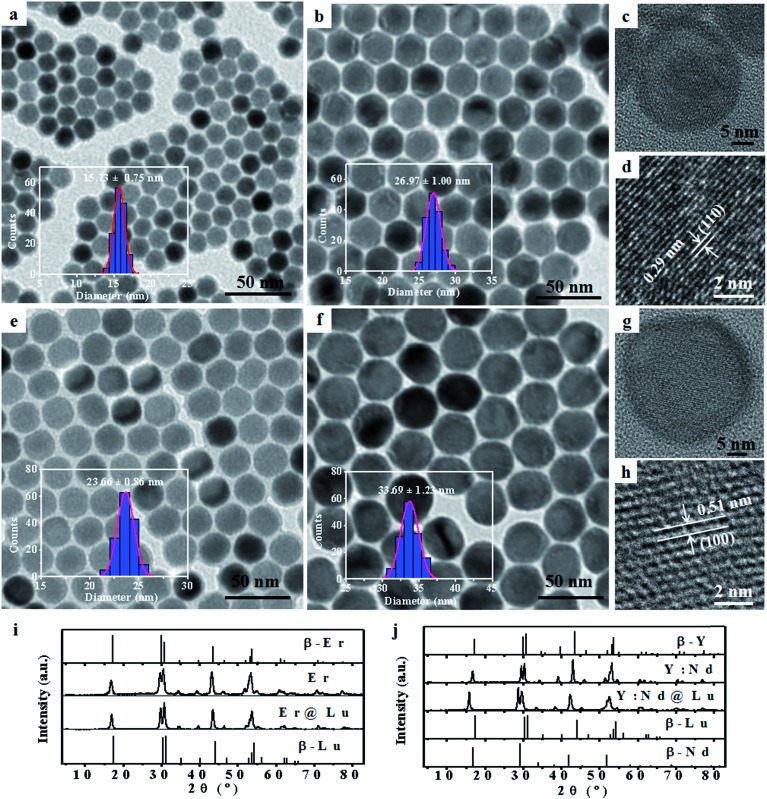
TEM images and size distribution (inset) of Er (a), Er@Lu (b), Y:Nd (e) and Y:Nd@Lu (f); HRTEM images of Er@Lu (c) and Y:Nd@Lu (g); lattice images of Er@Lu (d) and Y:Nd@Lu (h); PXRD patterns of Er and Er@Lu (i), Y:Nd and Y:Nd@Lu (j) and the JCPDS standard card of the pure hexagonal phase for NaErF_4_ (β-Er, 27-0689), NaLuF_4_ (β-Lu, 27-0726), NaYF_4_ (β-Y, 16-0334) and NaNdF_4_ (β-Nd, 27-0756).

Lanthanide fluoride has low phonon energy, high photostability, and good biocompatibility; thus, it is an excellent photoluminescence matrix for orthogonal SWIR emission.^25^,^31^,^32^ Firstly, NaErF_4_ (Er) and NaYF_4_:Nd (Y:Nd) core nanoparticles were synthesized by a previously reported solvothermal method.^33^ Transmission electron microscopy (TEM) images of Er (15.73 ± 0.75 nm) and Y:Nd (23.66 ± 0.86 nm) core nanoparticles were shown in Fig. 1a, e and S1†. NaLuF_4_ was selected as the shell due to its wide energy gap, low phonon energy, matched crystal lattice for passivating the surface of core nanoparticles and enhancing the SWIR emission.^32^ Furthermore, NaLuF_4_ could form a uniform shell on the surface of the core nanoparticles, which resulted in the formation of uniform sphere nanoparticles.^34^ The synthesized core nanoparticles, which acted as seeds, then underwent another solvothermal reaction to form core–shell nanoparticles.^35^ The two core–shell nanoparticles, NaErF_4_@NaLuF_4_ (Er@Lu) and NaYF_4_:Nd@NaLuF_4_ (Y:Nd@Lu), were synthesized by the seed-growth method. The TEM images of Er@Lu (26.97 ± 1.00 nm) and Y:Nd@Lu (33.69 ± 1.23 nm) were depicted in Fig. 1b, f and S2†. All inserted figures showed that the synthesized nanoparticles had a narrow size distribution (Fig. 1a, b, e, and f), and these data were summarized in Table S1.† The NaLuF_4_ shell thickness was approximately 5 nm which was the optimal shell thickness in the reported reference.^36^ The high resolution TEM (HRTEM) images of a single nanoparticle showed that good crystal stability was achieved using this method of synthesis, where an accelerating voltage of 200 kV was used (Fig. 1c and g). The lattice images showed that the interplanar crystal spacings of Er@Lu and Y:Nd@Lu were 0.29 and 0.51 nm (Fig. 1d and h), which matched the interplanar spacing of the (110) and (100) faces of pure hexagonal phase NaLuF_4_, respectively. The selected area electron diffraction (SAED) diagrams showed that the synthesized core–shell nanoparticles had a multi-crystal electron diffraction pattern of pure β-phase NaLuF_4_ (Fig. S3†). The powder X-ray diffraction (PXRD) pattern was used to further assess the phase state of the synthesized nanoparticles, which matched the standard pure hexagonal phase (Fig. 1i and j). These results confirmed that all synthesized nanoparticles had a narrow size distribution and were a pure β-phase.

**Fig. 2:**
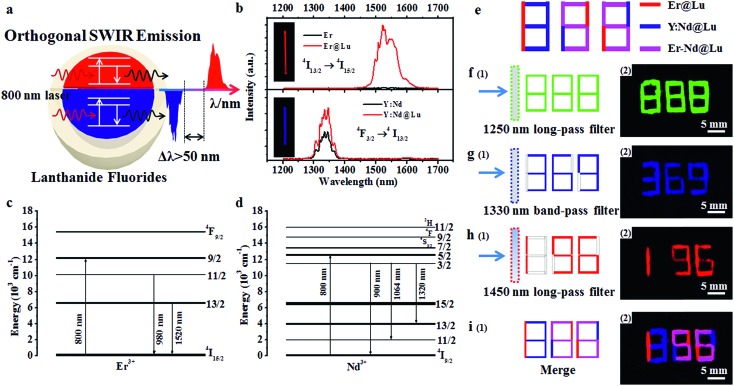
(a) Scheme of the orthogonal SWIR emission of core–shell structure lanthanide fluoride; (b) SWIR spectra of Er, Er@Lu, Y:Nd and Y:Nd@Lu excited by an 800 nm laser; Energy level diagram and the corresponding transition of Er^3+^ (c) and Nd^3+^ (d); schematic diagram of designed SWIR numeric codes (e); scheme and corresponding SWIR imaging pseudo-color photos with a 1250 nm long-pass filter (f), 1330 nm band-pass filter (g), 1450 nm long-pass filter (h) and after being merged *in situ* by software (i), which used Er@Lu (red), Y:Nd@Lu (blue) and a mixture cyclohexane dispersion of Er@Lu and Y:Nd@Lu (pink).

The SWIR spectra were obtained with an equipped external continuous laser (Fig. 2a). The Er and Y:Nd nanoparticles emitted SWIR light that was centered at 1522 and 1334 nm (Fig. 2b), which originated from a transition from the excited state ^4^I_13/2_ to the ground state ^4^I_15/2_ of Er^3+^ and the excited state ^4^F_3/2_ to the excited state ^4^I_13/2_ of Nd^3+^, respectively (Fig. 2c and d).^28^,^37^ In order to enhance the SWIR emission, the NaLuF_4_ shell was introduced, where the 1334 and 1522 nm emissions were 1.7 and 41.5 times higher than those of the core nanoparticle, respectively (Fig. 2b). This enhancement was ascribed to the surface passivation of core nanoparticles, where the high-energy excited state on the surface was easily trapped and dissipated by a nonradioactive transition as heat; thus, an enhanced SWIR emission was obtained after passivation of the surface and mitigation of the trapped state by the passivated shell NaLuF_4_.^34^,^36^,^38^ This mechanism was further supported by the upconversion luminescence (UCL) spectra of Er and Er@Lu, where the core–shell nanoparticles emitted a bright UCL signal, but barely UCL signals were found in core nanoparticles (Fig. S4†).^34^ Moreover, it was found in both Er@Lu and Y:Nd@Lu that the SWIR emission intensity excited by the 800 nm laser was enhanced by 1.3 times compared with that at 808 nm (Fig. S5b†), as Er^3+^ and Nd^3+^ had higher absorbance values at 800 nm. Furthermore, the distinction of emission intensity between Y:Nd@Lu and Er@Lu was mitigated by simply adjusting the concentration (Fig. S5c and d†). In addition, spectra overlap was observed between the emission of Y:Nd@Lu (1008–1126 nm, ^4^F_3/2_ → ^4^I_11/2_) and Er@Lu (962–1014 nm, ^4^I_11/2_ → ^4^I_15/2_) in the near infrared region (Fig. S5a†). It could be seen that the separation distance of SWIR emission spectra between Er@Lu (1448–1656 nm) and Y:Nd@Lu (1290–1384 nm) was more than 50 nm; thus, the SWIR signals showed noninterference and could be further applied in various fields. It could be hypothetic that the SWIR emission of both Er^3+^ and Nd^3+^ could be realized in the single nanoparticles (Fig. S6a†), however, the SWIR emission signals of Er^3+^ and Nd^3+^ would not be independent on each other to form the orthogonal SWIR system (Fig. S6b–d†). These results confirmed that the SWIR emission was orthogonal between Y:Nd@Lu and Er@Lu in nature.

It is known that the orthogonal emission can be separated by special optical filters to obtain a single emission signal. To confirm this, fiber filter paper (width × length: 1 × 5 mm) was dipped in Er@Lu, Y:Nd@Lu or a mixture containing Er@Lu and Y:Nd@Lu (Er–Nd@Lu) cyclohexane dispersion solution, and then dried naturally by solvent volatilization. The special array diagram, which was used for the following numeric information identification, was obtained by arranging size-specific filter paper according to the schematic diagram in Fig. 2e. The SWIR signals of Er@Lu or Y:Nd@Lu were collected by an NIR camera with an 800 nm laser as the excitation source, and the obtained images were shown in Fig. 2f–i after adding pseudo-color. When a 1250 nm long-pass filter (1250 filter) that permitted the emission of both Er@Lu and Y:Nd@Lu was used, the green Arabic number 888 was obtained (Fig. 2f-2), which accorded with the original design diagram (Fig. 2f-1). The Y:Nd@Lu emission was obtained using a 1330 nm band-pass filter (1330 filter) which only permitted the emission of Y:Nd@Lu to pass through, and the blue Arabic number 369 was obtained (Fig. 2g). The emission of Er@Lu was separately acquired using a 1450 nm long-pass filter (1450 filter), where the red Arabic number 196 was obtained (Fig. 2h). Furthermore, the individual SWIR pseudo-color images were merged *in situ* to achieve a new pseudo-color image (Fig. 2i-2), which clearly showed the location of Er–Nd@Lu by a distinct color and further demonstrated that the SWIR emission was completely separated by the optical filters without signal overlaps. These findings confirmed that SWIR emissions from Er@Lu and Y:Nd@Lu were orthogonal and can be applied in many fields, such as information encryption, anti-counterfeit, and *in vivo* bio-imaging.

Information encryption and anti-counterfeit play a vital role in personal identity verification, goods identification and security.^5^ Codes are used to mark production and protect goods against fakes.^39^,^40^ SWIR is invisible to the naked eye, which means that SWIR has a higher security level than other optical codes, and has widened the range to permit greater information capacity and more complex decoding processes to prevent illegal copying, which would permit a special scenario with higher safety.

**Fig. 3:**
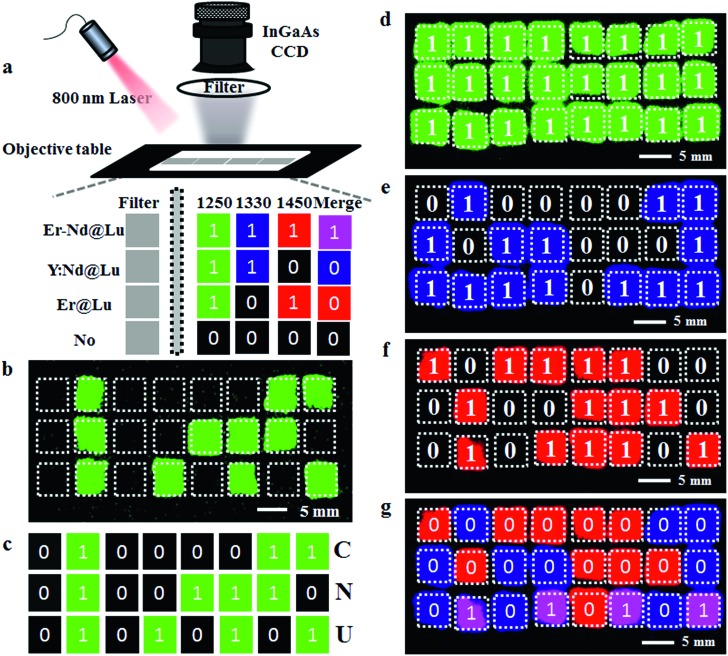
(a) Scheme of an invisible SWIR logical code, and “OR” and “AND” logical operations with optical filters; (b) SWIR image collected by a 1250 filter of a 3 × 8 array containing Er@Lu, Y:Nd@Lu and Er–Nd@Lu and the corresponding SWIR logical code (c); SWIR images collected by a 1250 filter (d), 1330 filter (e) and 1450 filter (f) of a 3 × 8 array containing Er@Lu, Y:Nd@Lu and Er–Nd@Lu and an “AND” logical operation (g) between images e and f.

As the invisible SWIR emissions of Er@Lu and Y:Nd@Lu were distinguished using optical filters and their cyclohexane dispersion was approximately transparent,^40^ we assumed that these two nanoparticles could be used in the invisible SWIR logical code and in information encryption. The operation mode of a logical code was shown in Fig. 3a, and two logical states “1” and “0” were also defined by the presence and absence of an SWIR signal, respectively. Firstly, the prepared square paper (5 × 5 mm), which was soaked in a cyclohexane dispersion of Er@Lu, Y:Nd@Lu or Er–Nd@Lu and then dried naturally, was arranged to form 3 × 8 arrays for the following invisible logical code and operation. The SWIR imaging pseudo-color image with a 1250 filter was obtained (Fig. 3b), where each green signal area was “1”, and the other area was “0”. Furthermore, the secret information “CNU” was obtained according to the American Standard Code for Information Interchange (ASCII, Fig. 3c). Next, the logical coding ability of the orthogonal SWIR emission was investigated, which was also the information coding ability. Three SWIR imaging pseudo-color images were collected by 1250 (Fig. 3d), 1330 (Fig. 3e), and 1450 filters (Fig. 3f), which were processed into a binary logical code according to the above-mentioned definition (Fig. 3a). Logical operation was then introduced to construct an integrated SWIR logical coding system, where logical “AND” and “OR” operations were investigated. Firstly, the acquired SWIR pseudo-color images with 1330 and 1450 filters (Fig. 3e and f) were merged *in situ* to obtain a new image, where a new color (pink) only emerged in the presence of both Er@Lu and Y:Nd@Lu (Fig. 3g). Secondly, the new image was processed into a new logical code using the principle that the pink was “1” and the other was “0”. It was found that the new logical code coincidentally matched the logical code directly produced by the “AND” logical operation according to the definition (Table S2†). This result confirmed that the “AND” logical operation could be achieved in the invisible SWIR logical code. The logical “OR” operation was similar to the “AND” logical operation, where the obtained new code showed all “1” logical states (Fig. 3d). Furthermore, the analogical approach produced by logical operations “NOT” (b and g), “OR” (c), “NOR” (h), “AND” (d), “NAND” (i), “XNOR” (e) and “XOR” (j), which were all obtained by the basic logical code (a and f), are shown in Fig. S7.† These results demonstrated that the SWIR logical code could be operated by the basic logical operations “AND”, “OR” and “NOT”, and more complex logical operations.

**Fig. 4:**
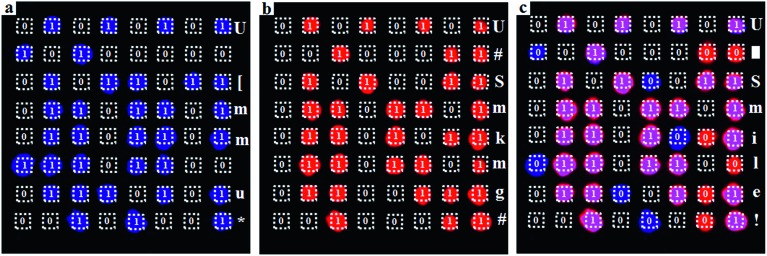
Invisible SWIR logical code for the information (“U Smile!”) encryption experiment; SWIR pseudo-color images, and logical coding and corresponding decoding information of single Nd^3+^ emission (a), single Er^3+^ emission (b) and “AND” logical operation (c).

Next, the SWIR logical code was explored in the invisible information encryption and anti-counterfeit fields. Firstly, the information “U Smile!” was transformed into a logical code according to ASCII, which was subsequently coded by the “AND” logical operation. Then, the precise position on the paper was dipped in the corresponding materials and quickly dried by heating. A distinct difference was not found between the dipped area and the blanks in the images on the paper (Fig. S8†), which showed that the information “U Smile!” was hidden under the conventional conditions. Secondly, the two individual logical codes collected by an NIR camera were decoded into information, according to ASCII. However, insignificant information was acquired, which further confirmed that the information was hidden (Fig. 4a and b). The individual SWIR pseudo-color images were then merged *in situ* to obtain a new image, which produced a new logical code using the logical “AND” operation, where pink was “1” and the other was “0”. The newly acquired logical code was decoded into information “U Smile!” according to ASCII (Fig. 4c). This result confirmed that the correct information “U Smile!” was acquired only by the logical “AND” operation, and not by other logical operations. The hidden encrypted information was coded and decoded by the orthogonal SWIR logical code and the corresponding logical operation, which was confirmed by these results. Furthermore, it could also form a more complex hidden code according to the various logical operations (Table S2†), which could code more complicated information and be used in hidden anti-counterfeit codes and special identity authentication.

**Fig. 5:**
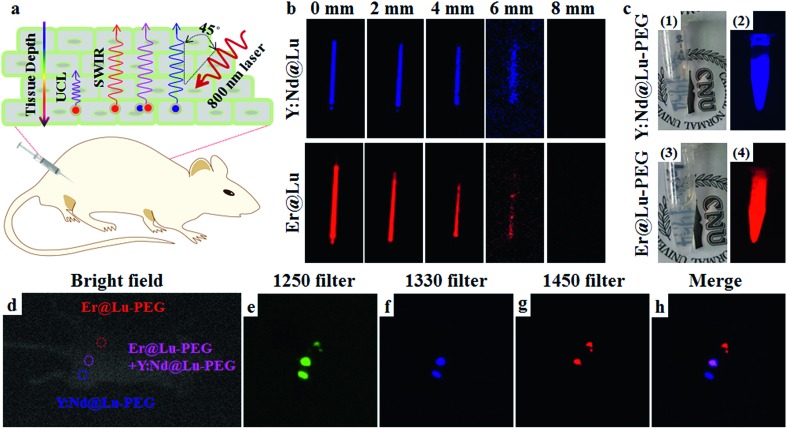
(a) Scheme of orthogonal SWIR imaging *in vivo*; (b) SWIR imaging pseudo-color photos of a glass capillary tube containing Y:Nd@Lu or Er@Lu (10 mg mL^–1^) excited by an 800 nm laser (0.114 W cm^–2^) under beef tissue of various thicknesses; practice photos (c1 and c3) and SWIR imaging pseudo-color photos (c2 and c4) of the Y:Nd@Lu–PEG or Er@Lu–PEG aqueous dispersion; bright field photos (d) and SWIR imaging pseudo-color photos with 1250 (e), 1330 (f), and 1450 (g) filters collected by an NIR camera and merged pseudo-color photos (h) *in situ* from (f) and (g).

SWIR imaging *in vivo* has the advantage of fast imaging, and it is inexpensive and highly sensitive, and it has a higher spatial resolution and tissue penetration depth than visible light.^10^ Firstly, a tissue mimetic experiment of SWIR imaging was carried out, where a glass capillary tube containing Er@Lu or Y:Nd@Lu was covered by beef tissue of various thicknesses. The degree of the angle between the incidence directions of the 800 nm laser and signal direction was approximately 45° (Fig. 5a). This result showed that the SWIR signal gradually weakened until it was no longer detected by the NIR camera when the beef tissue thickness was approximately 8 mm (Fig. 5b). However, the UCL of Er@Lu was not detectable under 4 mm beef tissue, which confirmed that higher tissue penetration was achieved with SWIR imaging (Fig. S9†). Recently, many researchers have reported SWIR imaging *in vivo*, using various materials. These studies primarily focus on “single-color” imaging *in vivo*.^10^–^13^ Achieving multi-color orthogonal SWIR imaging *in vivo* is still a challenge.^9^ Orthogonal SWIR emission has no signal overlap in nature. Therefore, interference in orthogonal SWIR emission can be eliminated and the accuracy of signal identification is increased. Therefore, it is meaningful to further investigate the application of orthogonal SWIR emission in imaging *in vivo*, which can result in an orthogonal SWIR imaging mode *in vivo*.

Firstly, hydrophobic nanoparticles were prepared using the solvothermal method as imaging probes *in vivo*, and hydrophilic nanoparticles were then obtained from the hydrophobic nanoparticles by surface modification.^41^ Naked nanoparticles were acquired by removing the OA ligand using an acidic aqueous solution.^42^ Then, PEG-modified nanoparticles (Er@Lu–PEG and Y:Nd@Lu–PEG) were obtained by mixing the naked nanoparticles with PEG–COOH, which was confirmed by FTIR and transformation of the surface charge from positive to negative (Fig. S10 and Table S3†). The DLS measurements showed that both Er@Lu–PEG and Y:Nd@Lu–PEG with a similar layer thickness had an effective hydrodynamic diameter (Fig. S11†), which further illustrated good dispersion in water (Fig. 5c1 and c3). Irradiated by an 800 nm laser, Er@Lu–PEG and Y:Nd@Lu–PEG still emitted bright SWIR signals (Fig. 5c2 and c4). The above results confirmed that the hydrophilic nanoparticles had good aqueous dispersion and SWIR emission properties, which further verified their potential application in the bio-imaging field. Secondly, the biocompatibility of Er@Lu–PEG and Y:Nd@Lu–PEG was assessed by the MTT assay, where the obtained results showed that the relative cell viability of the HCT116 cell line in all groups was greater than 85% following incubation for 12 or 24 hours with various nanoparticle concentrations ranging from 0 to 1.2 mg mL^–1^ (Fig. S12†). The biocompatibility of Er@Lu–PEG and Y:Nd@Lu–PEG was confirmed and they were used as biomaterials for imaging *in vivo*, and the SWIR imaging capacity *in vivo* was then measured in an animal model.^43^,^44^

All *in vivo* experiments obeyed the rules and operational norms of the Institutional Animal Care and Use Committee (IACUC). To further demonstrate that these two nanoparticles could achieve orthogonal SWIR imaging *in vivo*, a nude mouse was subcutaneously injected with Er@Lu–PEG, Y:Nd@Lu–PEG or a mixture of Er@Lu–PEG and Y:Nd@Lu–PEG (Er–Nd–PEG) aqueous dispersion at three positions marked in the bright field image (Fig. 5d), which was constructed for performing SWIR imaging *in vivo*. The three injection points were clearly shown by SWIR imaging with a 1250 filter (Fig. 5e). Individual SWIR imaging *in vivo* with a 1330 or 1450 filter demonstrated that no signal interference in the area injected with single Y:Nd@Lu–PEG or Er@Lu–PEG was found (Fig. 5f and g), and the position and shape of the signal area matched those of the 1250 filter, respectively. These results confirmed that orthogonal SWIR imaging *in vivo* was achieved without signal interference. Moreover, Fig. 5h was merged *in situ* with Fig. 5f and g, and the Er–Nd–PEG injection point showed a new color (pink), which supported the colocalization ability of orthogonal SWIR imaging *in vivo*. SWIR imaging in a tumor-bearing mouse, which was obtained after intravenously injecting the Y:Nd@Lu–PEG and Er@Lu–PEG mixture aqueous dispersion, accorded with the result obtained by direct subcutaneous injection (Fig. S13†). Therefore, orthogonal SWIR imaging *in vivo* could be expected to improve the simultaneous imaging capacity for various diseases or enhance the accuracy of disease diagnosis by colocalization, which may be achieved in the future.^45^

## Conclusions

In summary, this work demonstrated that the SWIR emissions of Er@Lu and Y:Nd@Lu excited by an 800 nm laser were completely separated by optical filters and orthogonal. The orthogonal SWIR emission could be further used for an invisible logical code by manipulating the emission signal of nanoparticles. These properties of the synthesized nanoparticles can be further applied in invisible information encryption and anti-counterfeit fields. Furthermore, SWIR imaging *in vivo* also demonstrated that the synthesized hydrophilic Er@Lu–PEG and Y:Nd@Lu–PEG were capable of orthogonal SWIR imaging *in vivo* without signal interference. We believe that this orthogonal SWIR emission mode may provide innovative insights into the code for invisible information encryption, anti-counterfeit and multi-color orthogonal bio-imaging *in vivo*.

The animal experiment in this work strictly abided by the regulations of Institutional Animal Care and Use Committee (IACUC). The nude mice in this experiment were provided by Beijing Vital River Laboratory Animal Technology Co., Ltd. And all experiments followed institutional guidelines and were performed in compliance with relevant laws, and approved by the Animal Ethics Committee of the Vital River Institutional Animal Care and Use Committee (VR IACUC).

## Conflicts of interest

There are no conflicts of interest to declare.

## Supplementary Material

Supplementary information
