# Supplementary material for: Orthogonal shortwave infrared emission based on rare earth nanoparticles for interference-free logical codes and bio-imaging
Source: Chem Sci. 2019 Jan 24;10(11):3281–8. doi: 10.1039/c8sc05044a (PMC6429594; doi:10.1039/c8sc05044a)
Supplement: Supplementary file 1 [file SC-010-C8SC05044A-s001.pdf]

Electronic Supplementary Material (ESI) for Chemical Science.
This journal is © The Royal Society of Chemistry 2019

Supporting Information

Orthogonal Shortwave Infrared Emission Based on Rare Earth Nanoparticles for Interference-free Logical Code and Bio-imaging

By *Liyi Ma,* [a, b, c, §] *Xuejiao Zhai,* [a, §] *Gaiping Du,* [b, c] and *Jing Zhou**[a]

[a] Department of Chemistry, Capital Normal University, Beijing 100048, People's Republic of China

[b] Key Laboratory of Photochemical Conversion and Optoelectronic Materials, Technical Institute of Physics and Chemistry, Chinese Academy of Sciences, Beijing 100190, People's Republic of China

[c] University of Chinese Academy of Sciences, Beijing 100049, People's Republic of China

E-mail: jingzhou@cnu.edu.cn

KEYWORDS: Orthogonal shortwave infrared, SWIR logical codes, anti-counterfeit, bio-imaging, lanthanide fluoride nanoparticles

Experiment section

Chemicals and materials: Yttrium (III) chloride hexahydrate ($YCl_3{\cdot}6H_2O$, 99.99%), erbium (III) chloride hexahydrate ($ErCl_3{\cdot}6H_2O$, 99.99%), neodymium (III) chloride hexahydrate ($NdCl_3{\cdot}6H_2O$ 99.99%), lutetium (III) chloride hexahydrate ($LuCl_3{\cdot}6H_2O$, 99.99%) were all purchased from Shanghai Energy Co., Ltd. Oleic acid (OA, 90%) was purchased from Sigma-Aldrich Co., Ltd. Ammonium fluoride (NH_4F, 98%) and 1-octadecene (ODE, 90%) were provided by Alfa Aesar Co., Ltd. Ethanol, cyclohexane, sodium hydroxide (NaOH, ≥96%), diethyl ether, and acetone were purchased from Beijing Shiji Co., Ltd. Hydrochloric acid (HCl) was supported by Sinopharm Chemical Reagent Co., Ltd. Methanol anhydrous

(CH_3OH, ≥99.9%) was provided by Tianjin Fuchen Chemical Reagent Co., Ltd. 3-(4,5-Dimethylthiazol-2-yl)-2,5-diphenyltetrazolium bromide (MTT) and dimethyl sulfoxide (DMSO) were provided by BioRuler Co., Ltd. The methoxy(polyethylene glycol)-2000-COOH (PEG-COOH) was purchased from Beijing Wande technology Co., Ltd. All of the chemical reagents were directly used without further purification unless otherwise noted. And the deionized (DI) water was used throughout the experiment.

Characterization: Powder X-ray diffraction (PXRD) patterns of the products were collected in the 2θ from 5 to 85 at the scanning rate of 4 min^{-1} by using Rigaku DMAX 2500 diffractometer which equipped with a Cu Kα radiation (λ = 1.5406 Å) at 36 kV tube voltages and 20 mA tube currents. The transmission electron microscopy (TEM) images were got by preparing copper girds with directly drying a drop of dilute cyclohexane dispersion of nanoparticle which were observed with Hitachi-7650 transmission electron microscope under the condition of 80 kV voltages. High-resolution transmission electron microscope (HRTEM) images and selected area electron diffraction (SAED) images were acquired from JEOL 2100F transmission electron microscopy with 200 kV operation voltages. NIR fluorescence emission spectra of the nanoparticle with the concentration of 10 mg mL^{-1} or 0.03 M were obtained by FL920 spectrometer (Edinburgh Instruments Ltd.) under the excitation of external 800 or 808 nm continuous laser (Changchun Weimi Electronic Instrument Co., Ltd). The SWIR imaging *in vitro* and *in vivo* were acquired with the optical filters by the Princeton NIR Vana640 camera under an 800 nm continuous laser. The upconversion luminescence (UCL) spectra were gained by FL7000 fluorescence spectrophotometer (Hitachi) with equipping an external 800 nm laser as the excitation source. The dried nanoparticles and KBr were pressed to a thin slice by the infrared powder tablet, which was used to measure the nanoparticles' FTIR on a Bruker TENSOR 27 FTIR Spectrometer. The hydrodynamic diameter and Zeta potential were measured with high-performance particle sizer (HPPS, Malvern Instrument). To make the data of the size distribution measurement more accurate, aqueous dispersion (5

mg mL^{-1}) was filtered with the syringe filter (0.22 μm). Zeta potential of the nanoparticle aqueous dispersion was directly measured without the syringe filter.

Synthesis of OA-capped $NaErF_4$ (Er) and $NaYF_4$:5%Nd (Y:Nd) nanoparticles: The Er and Y:Nd nanoparticles were synthesized according to the previously reported solvothermal method.[33] Firstly, 1 mmol $ErCl_3·6H_2O$ and 3 mL anhydrous ethanol were added into 100 mL three-neck flask, which was transformed into transparent solution by sonication. Next, 6 mL OA and 17 mL ODE were injected into the flask. The mixture was slowly heated to 120 ℃ with vigorous stirring under vacuum to get clear transparent solution, then, to 160 ℃ for 5 minutes to remove trace water. After that the temperature was cooled down to room temperature, 8 mL methanol containing 4 mmol NH_4F and 2.5 mmol NaOH was added to above mixture, then heated to 50 ℃ for 1 hour with vigorously stirring. Next, the slurry was slowly heated to 110 ℃ for about 10 minutes to remove the methanol, water and oxygen. Afterward, the mixture was heated quickly to 305 ℃, and maintained for 1 hour under argon protection. After the reaction was finished and cooled down to room temperature, the product (Er) was collected by centrifugation and washed with cyclohexane and ethanol for three times. The nanoparticle (Y:Nd) was obtained using 0.95 mmol $YCl_3·6H_2O$ and 0.05 mmol $NdCl_3·6H_2O$ to replaced $ErCl_3·6H_2O$, where other synthesis procedure was same as the Er. Finally, the obtained Er or Y:Nd was dispersed in 10 mL cyclohexane.

Synthesis of OA-capped $NaErF_4$@$NaLuF_4$ (Er@Lu) or $NaYF_4$:5%Nd@$NaLuF_4$ (Y:Nd@Lu) core-shell nanoparticle: The synthesis of core-shell nanoparticle was similar as the Er with slight modification. 1 mmol $LuCl_3·6H_2O$, 3 mL ethanol, 6 mL OA and 17 mL ODE were added into 100 mL three-neck flask, which was slowly heated to 160 ℃ to remove ethanol and water, and to get transparent solution. Then 8 mL methanol containing 4 mmol NH_4F and 2.5 mmol NaOH, and 6 mL cyclohexane containing the prepared Er were added into above solution, which was heated to 50 ℃ for 1 hour, 110 ℃ for 10 minutes and 305 ℃ for 1 hour under argon protection. And the following experimental process of synthesis of

Y:Nd@Lu was similar as that of Er@Lu

Production of invisible SWIR logical code and data processing: Conventional fiber filter paper was tailored into square piece paper (5 mm × 5 mm). The pieces were soaked into cyclohexane dispersion of Er@Lu (10 mg mL^{-1}), Y:Nd@Lu (30 mg mL^{-1}), and mixture of Er@Lu and Y:Nd@Lu, respectively, and then dried naturally. Then the square piece paper containing materials were fixed on a big fiber paper to form array, where the array organization was according to the ASCII. The SWIR signal was collected by NIR camera with special optical filter and an 800 nm laser (0.114 W cm^{-2}) as excited source. The Arabic numerals array was similar to logical code, but strip paper (1 × 5 mm) substituted the square one. Grey-scale maps collected by NIR camera were colored by software ImageJ, where the signal obtained with 1250, 1330 and 1450 filter were marked by green, blue, and red, respectively. The signal overlap was carried out by merging *in situ* the two independent signals respectively acquired with 1330 and 1450 filters. Furthermore, the logical code was acquired, according to the definition of the two logical states "1" and "0" by the presence and absence of an SWIR signal. The quadrille paper was heated to 70 ~ 80 ℃ to accelerate the evaporation of cyclohexane, then the Er@Lu or Y:Nd@Lu was dropwise added into the small squares of paper, according to the code of information "U Smile!". The signal collection was similar to above description. The data processing and analysis were based on the software ImageJ.

Synthesis of hydrophilic $NaErF_4$@$NaLuF_4$-PEG (Er@Lu-PEG) or $NaYF_4$:Nd@$NaLuF_4$-PEG (Y:Nd@Lu-PEG) nanoparticle: To make the synthesized Er@Lu and Y:Nd@Lu nanoparticles used in biological field, the reported method was used for getting the hydrophilic nanoparticles. Firstly, 1 mL Er@Lu cyclohexane dispersion was mixed with the prepared HCl aqueous solution (pH = 3 ~ 4) and diethyl ether. The obtained mixed solution was repeatedly shocked and extracted until the phase interface between the aqueous phase and the oleic phase obviously appeared. And the aqueous phase was collected where the ligand-

free nanoparticles were recuperated by centrifugation after adding acetone as the precipitator. Lastly, the product was dispersed in 10 mL deionized water, and 60 mg PEG-COOH as the surfactant modifier was added into the above solution and stirred for 12 hours. Er@Lu-PEG was obtained by centrifugation and further dispersed in deionized water for using in following experiment. The synthesis of Y:Nd@Lu-PEG was similar to that of Er@Lu-PEG.

MTT assay: In order to make these nanoparticles to be applied in biological dimensions, it is essential to process experiments to evaluate the toxicity of the nanoparticles. The cytotoxicity *in vitro* was estimated by the typical reduction activity methyl thiazolyl tetrazolium (MTT) assay. Firstly, the HCT116 cells were cultured with culture medium in a 96-well plate at a density of 10^5 per well in incubator at the condition of 5% CO_2 and 37℃ for 12 hours. Then 100 μL of culture medium containing various concentrations of Er@Lu-PEG or Y:Nd@Lu-PEG (0, 0.1, 0.2, 0.4, 0.8, 1.2 mg mL^{-1}) were added into corresponding wells to replace the original one. After 12 or 24 hours later, the MTT (10 μL, 5 mg mL^{-1}) was added into each well and this plate was cultured for another 4 hours under the same conditions. Next, 100 μL dimethylsulfoxide (DMSO) was added into each well in this plate, and then this plate was placed in incubator for 30 minutes to dissolve the frozen crystals. Lastly, the absorbance value was measured by Tecan Infnite M200 monochromator-based multifunction microplate reader at the wavelength of 570 nm. The following formulate was used to access the cell viability:

Cell viability (%) = means of *Abs*. value of treatment group / *Abs*. value of control group × 100%

SWIR imaging *in vitro*: SWIR imaging signal of glass capillary tube (0.5 mm of inside diameter) containing Er@Lu or Y:Nd@Lu (10 mg mL^{-1}) was collected by Princeton NIR Vana640 camera (100 ms of exposure time) with 800 nm continuous laser (0.114 W cm^{-2}) as excitation source and a 1250 nm long-pass optical filter. The tissue mimics experiment of SWIR imaging *in vitro* was similar to above description, where the beef tissue of various thicknesses (provided by near store, the thickness was approximately 2 mm) covered on the

glass capillary. The 1.5 mL PE tube containing 1 mL aqueous dispersion of Er@Lu-PEG or Y:Nd@Lu-PEG was excited by 800 nm laser and the SWIR imaging pictures were acquired by an InGaAs based NIR camera with a 1250 nm long-pass optical filter, respectively. In the experiment design, the degree of angle between the incidence direction of excitation light (800 nm laser) and signal light collection direction was about 45 °. The tissue mimics experiment of UCL (Er@Lu) imaging *in vitro* was similar to that of SWIR imaging. The SWIR or UCL signal processing and analysis were based on the software ImageJ.

SWIR imaging *in vivo*: Before experiment, the nude mouse (body weight was about 20 g) was anesthetized with 50 μL of 10% chloral hydrate by introperitoneal injection under the authorization of regional ethic committee. The mouse was scanned by NIR camera before and after the hydrophilic nanoparticles was administrated. Then about 50 μL Er@Lu-PEG (5 mg mL^{-1}), Y: Nd@Lu-PEG (5 mg mL^{-1}) and the mixture of Er@Lu-PEG and Y:Nd@Lu-PEG aqueous dispersion were subcutaneously injected into the left crotch, right crotch and the middle of the two, respectively. And the SWIR imaging *in vivo* was obtained with the 1250, 1330 and 1450 nm optical filter by NIR camera, where the experiment setting was similar to that *in vitro*. The SWIR imaging for tumor colocalization was similar as that of subcutaneous injection. The tumor-bearing mouse model (body weight was about 20 g) was constructed by subcutaneously injecting 10^6 hela cell, which was not used for experiment until the tumor volume reached about 100 mm^3. The 200 μL of Y:Nd@Lu-PEG and Er@Lu-PEG mixture aqueous dispersion (10 mg kg^{-1}) were intravenously injected into tumor-bearing mouse body, followed by the SWIR imaging signals were collected after 45 minutes. All signal processing and analysis was based on the software ImageJ.

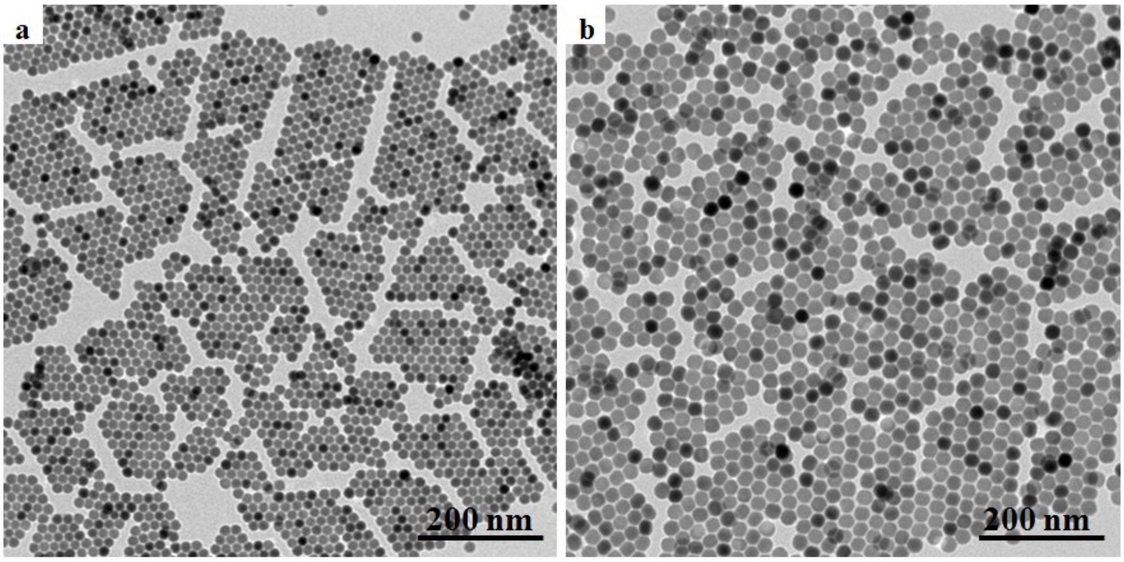

Figure S1. TEM images of Er (a) and Y:Nd (b).

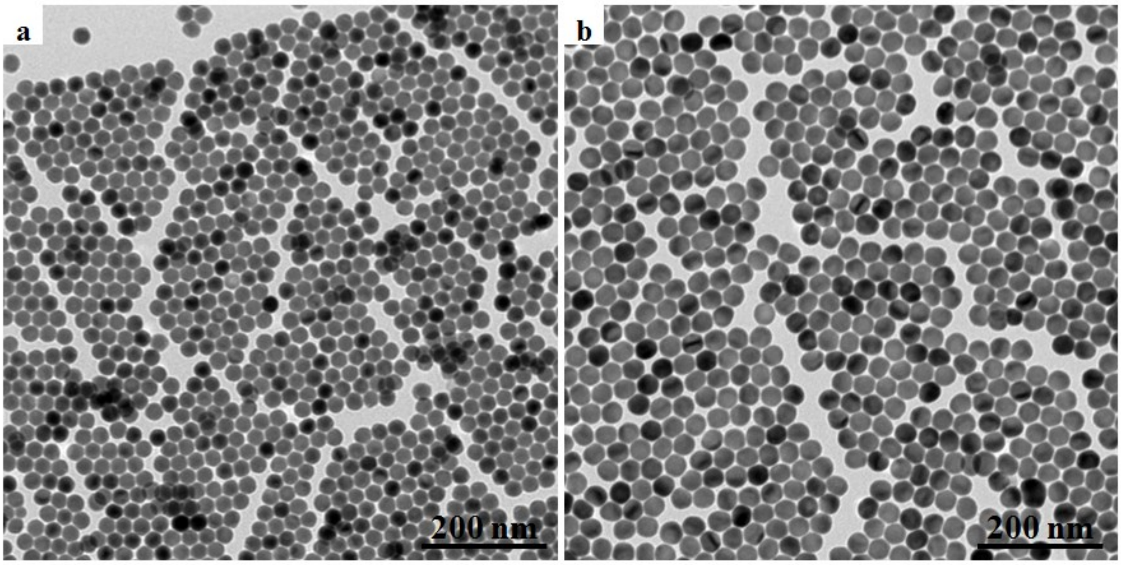

Figure S2. TEM images of Er@Lu (a) and Y:Nd@Lu (b).

Table S1. Size summary of synthesized nanoparticles

Materials	Core diameter (nm)	Relative deviation (%)	Core-shell diameter (nm)	Relative deviation (%)	Shell thickness (nm)
Er@Lu	15.73 ± 0.75	4.8	26.97 ± 1.00	3.7	5.62
Y:Nd@Lu	23.66 ± 0.86	3.6	33.69 ± 1.23	3.7	5.02

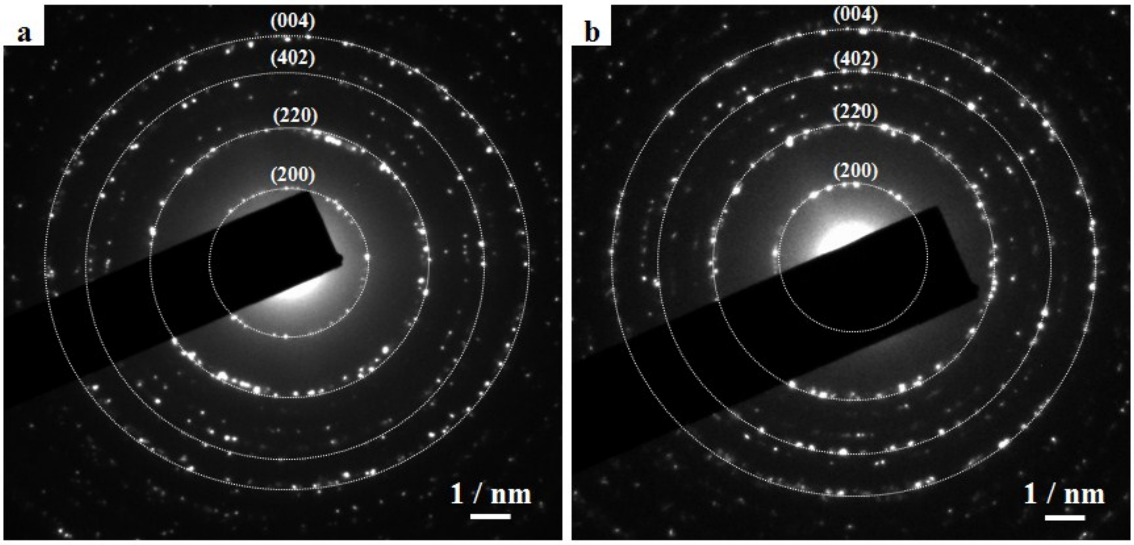

Figure S3. SAED diagram of Er@Lu (a) and Y:Nd@Lu (b).

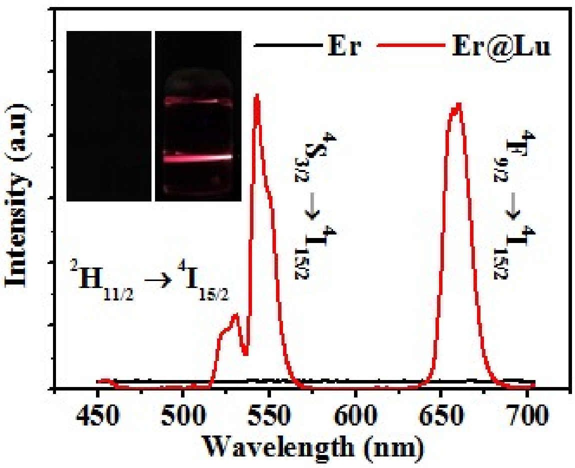

Figure S4. The UCL spectra and images (insert) of Er (left) and Er@Lu (right).

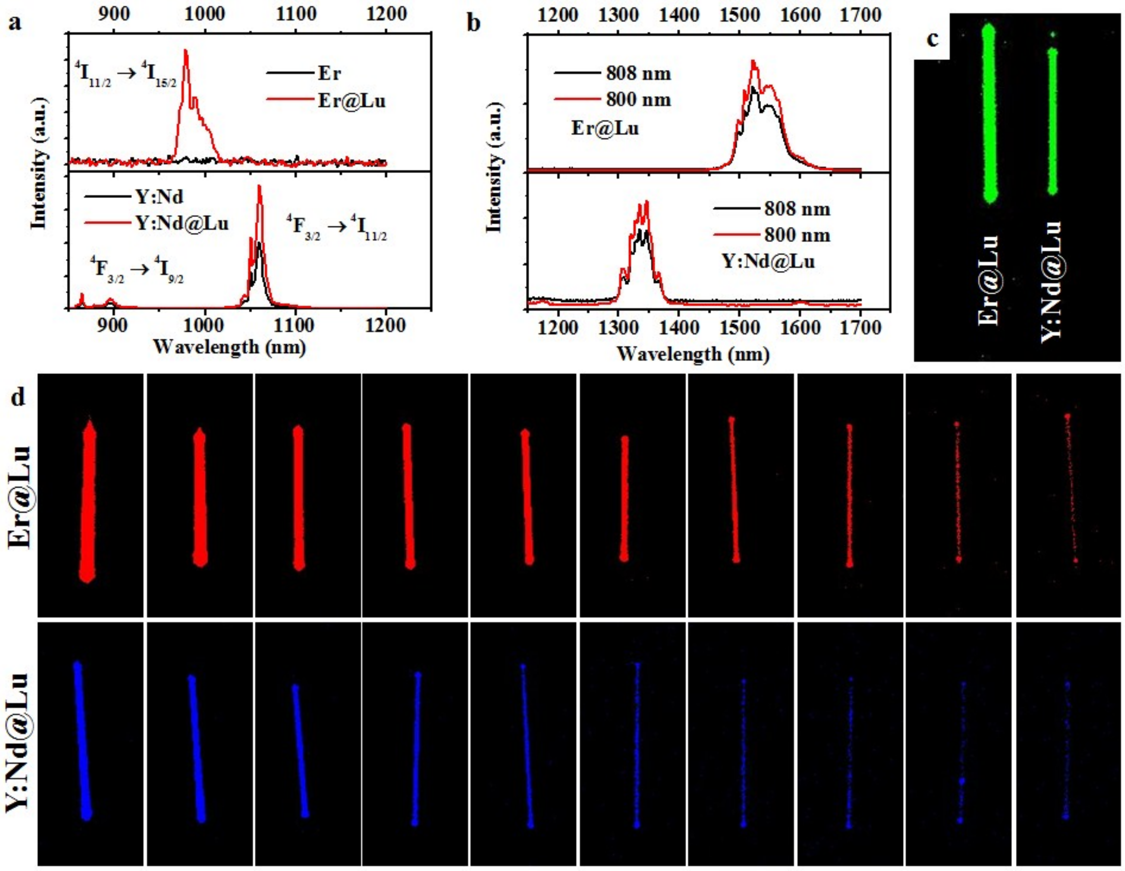

Figure S5. NIR spectra (a) of Er, Er@Lu and Y:Nd, Y:Nd@Lu excited by 800 nm laser. SWIR spectra (b) of Er@Lu (up) and Y:Nd@Lu (down) excited by 800 and 808nm laser, respectively; the SWIR imaging pseudo-color photos of Er@Lu and Y:Nd@Lu(c, 10 mg mL^{-1}); (d) SWIR imaging pseudo-color photos of various concentrations of Er@Lu (up) and Y:Nd@Lu(down).

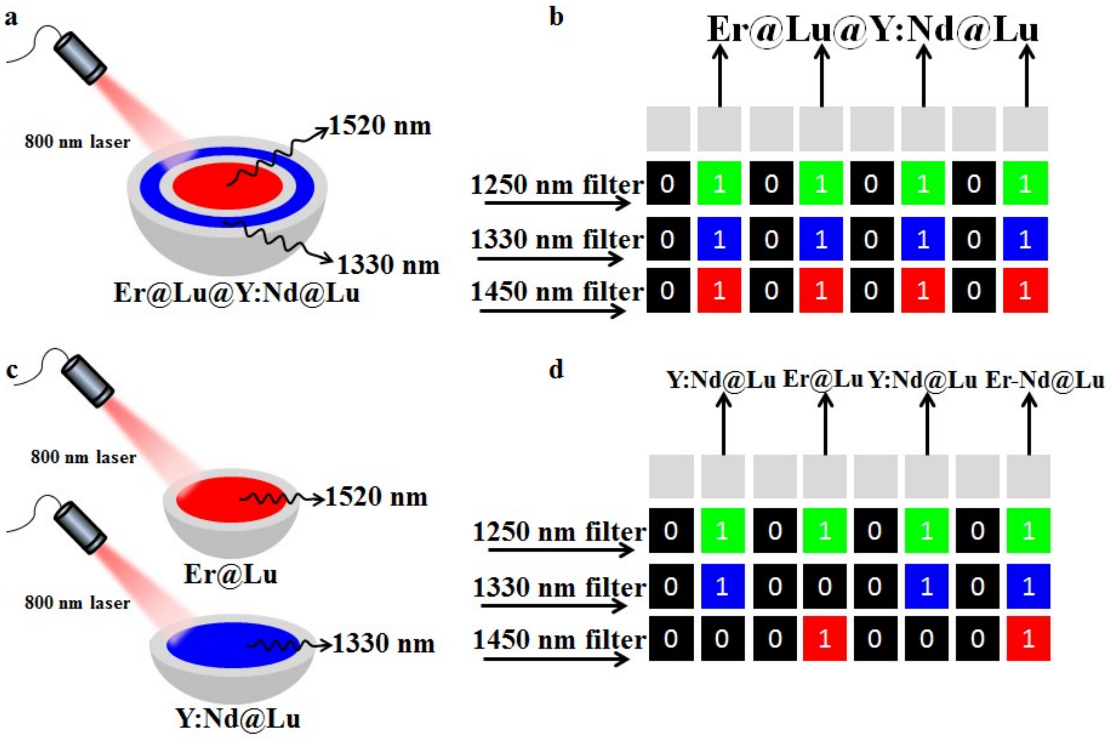

Figure S6. Scheme of SWIR emissions of $NaErF_4@NaLuF_4@NaYF_4:Nd@NaLuF_4$ nanoparticles (a) and corresponding logical operation (b), $NaErF_4@NaLuF_4$ and $NaYF_4:Nd@NaLuF_4$ nanoparticles (c) and corresponding logical operation (d) excited by 800 nm laser.

Table S2. Basic logical operation for logical codes

A	B	NOT(A)	NOT(B)	OR	AND	NAND	NOR	XOR	XNOR
1	1	0	0	1	1	0	0	0	1
1	0	0	1	1	0	1	0	1	0
0	1	1	0	1	0	1	0	1	0
0	0	1	1	0	0	1	1	0	1

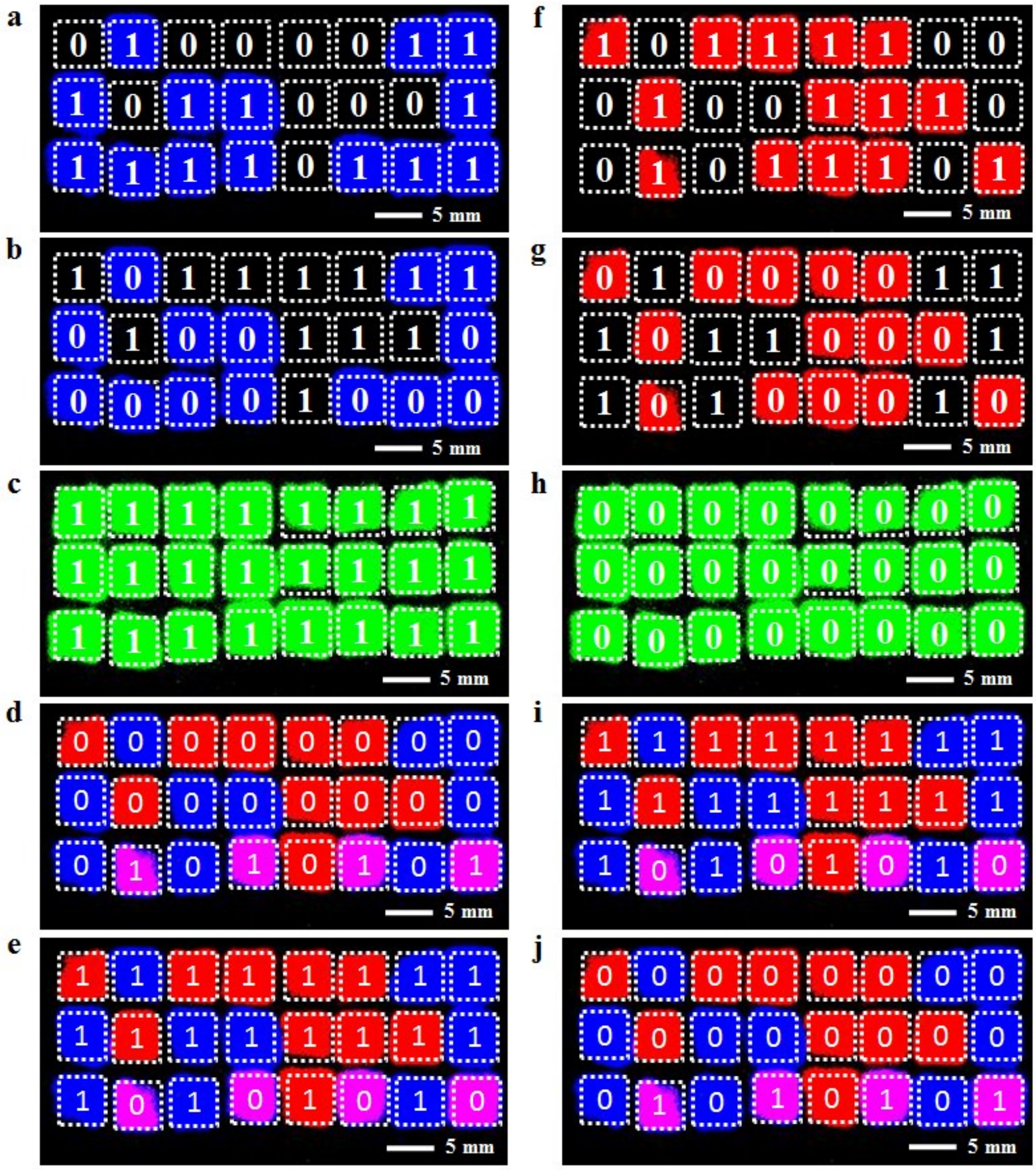

Figure S7. SWIR logical operation which used the Y:Nd@Lu (blue), Er@Lu (red) and mixture cyclohexane dispersion of Er@Lu and Y:Nd@Lu (pink merged from blue and red) for information by ASCII. SWIR logical code (a, f) got by logical definition whether the photoluminescence exist or not as “1” or “0”, respectively; “NOT” (b, g), “OR”(c), “NOR” (h), “AND” (d), “NAND” (i), “XNOR” (e), “XOR” (j) logical operations all formed from the definition of (a) and (f).

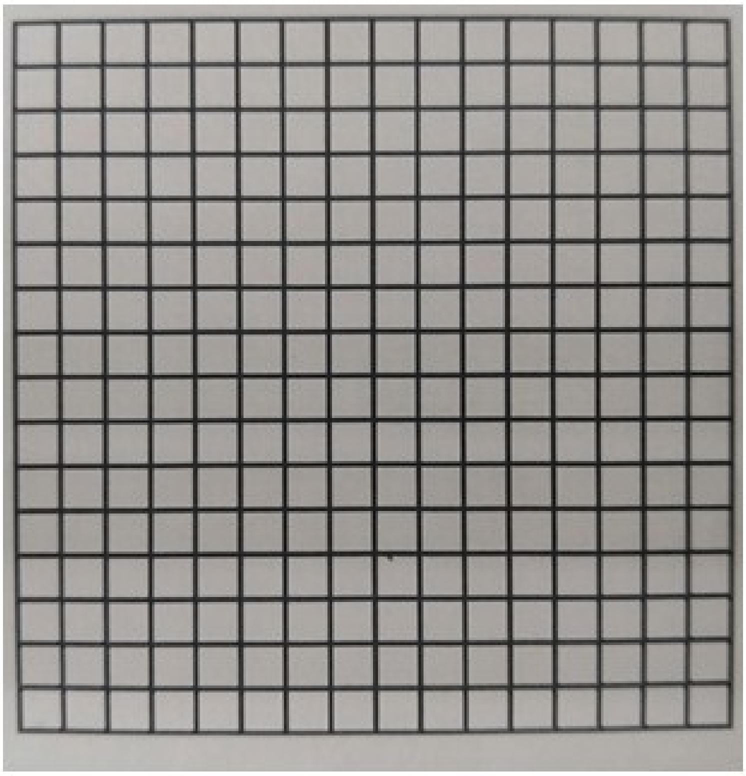

Figure S8. The practical image of Figure 4.

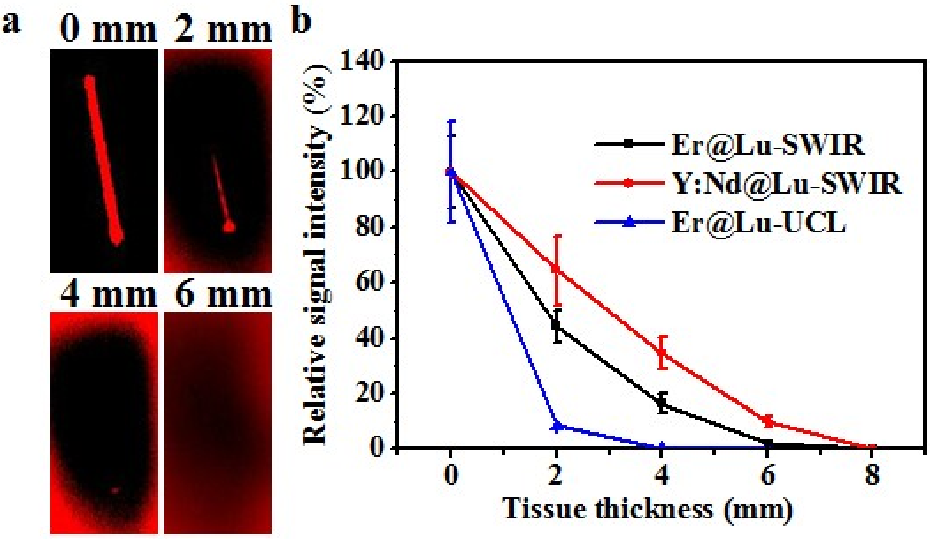

Figure S9. (a) UCL imaging pseudo-color images of glass capillary tube containing Er@Lu excited by 800 nm laser (0.114 W cm^{-2}) under beef tissue of various thicknesses; (b) relative signal intensity analysis of SWIR and UCL imaging.

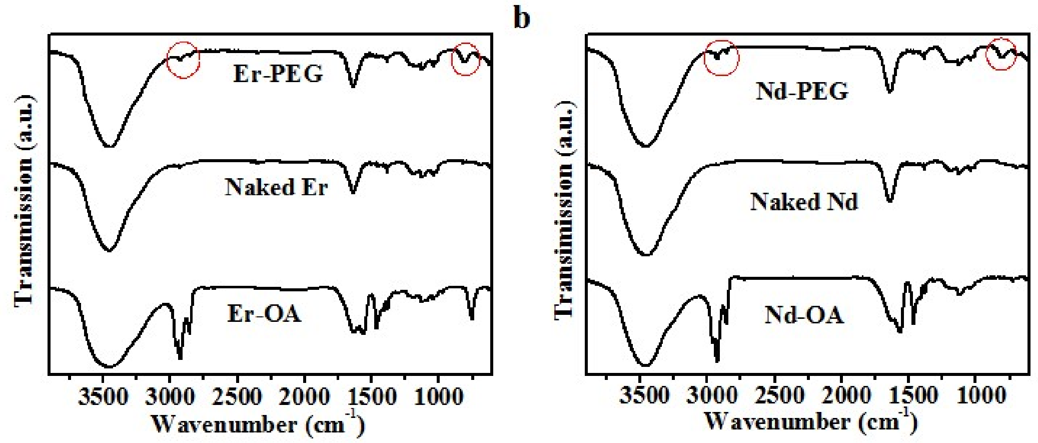

Figure S10. FTIR of OA-coated Er@Lu, naked Er@Lu, Er@Lu-PEG (a) and OA-coated Y:Nd@Lu, naked Y:Nd@Lu, Y:Nd@Lu-PEG (b).

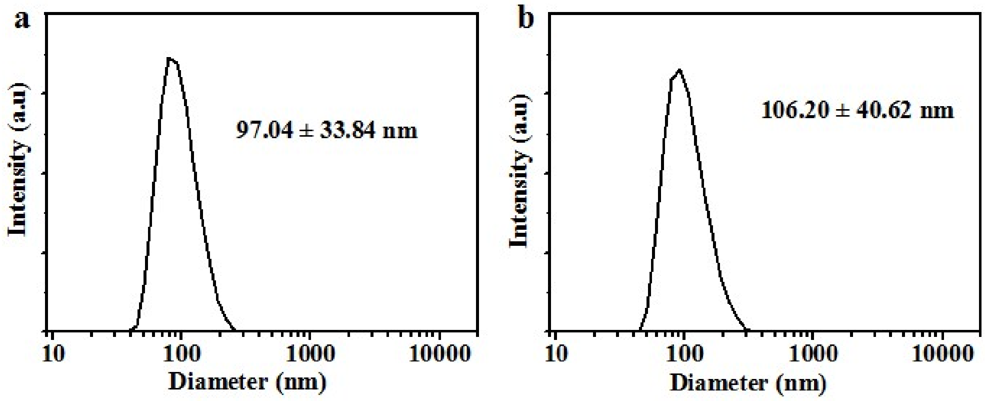

Figure S11. Hydrodynamic size distribution of Er@Lu-PEG (a) and Y:Nd@Lu-PEG (b).

Table S3. Zeta potential of the hydrophilic nanoparticles

	Naked Er@Lu	Er@Lu-PEG	Naked Y:Nd@Lu	Y:Nd@Lu-PEG
Zeta potential（mV）	29.2 ± 5.15	-5.589 ± 3.11	32.7 ± 6.36	-6.15 ± 4.06

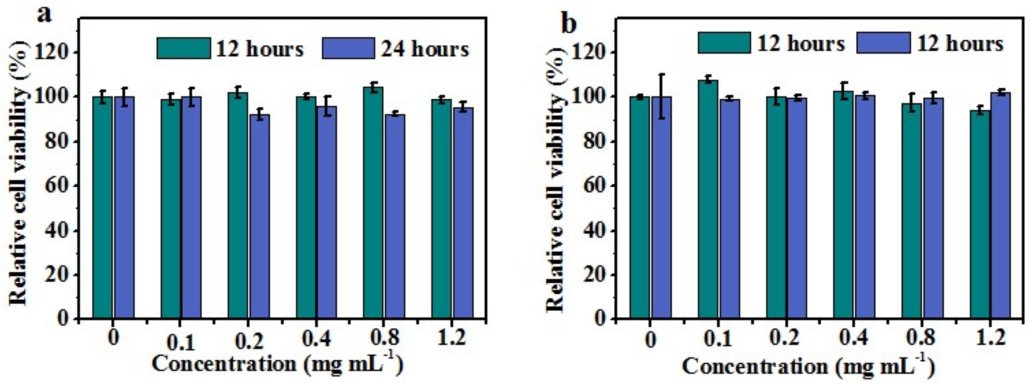

Figure S12. *In vitro* relative cell viability of HCT116 cell line incubated with Er@Lu-PEG (a) and Y:Nd@Lu-PEG (b) at various concentrations (0, 0.1, 0.2, 0.4, 0.8, 1.2 mg mL^{-1}) for 12 hours or 24 hours.

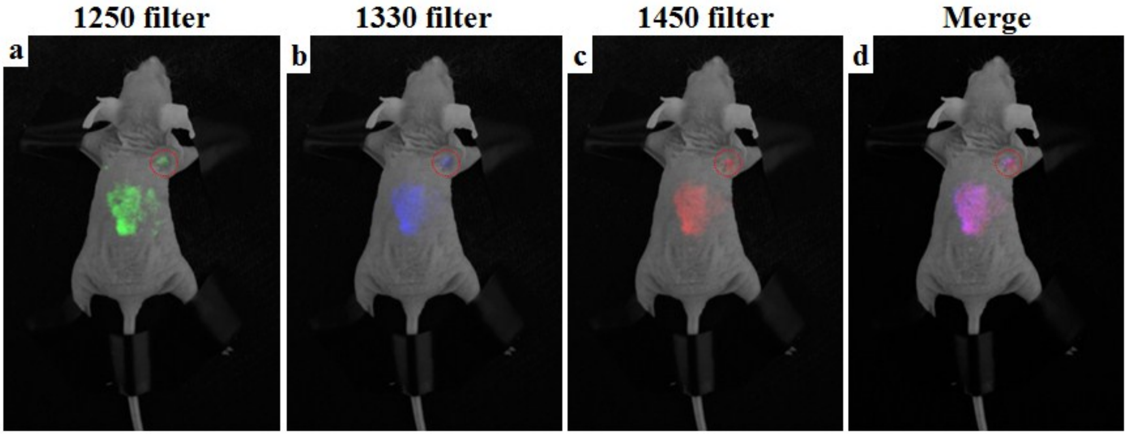

Figure S13. SWIR imaging pseudo-color photos of tumor-bearing mouse (tumor volume: 100 mm^3) after intravenous injection of Y:Nd@Lu-PEG and Er@Lu-PEG mixture aqueous dispersion (10 mg kg^{-1}) under 800 nm laser excitation (0.114 W cm^{-2}) collected with 1250 nm filter (a), 1330 nm filter (b), 1450 nm filter (c) and merged pseudo-color photos (d) *in situ* from (b) and (c). The tumor positions were marked by the red circle.
